# The intrinsic potential of IgY–polymyxin B nanocombinations for combating colistin-resistant *Salmonella enterica* serovar Typhimurium isolated from ready-to-cook chicken

**DOI:** 10.1016/j.psj.2026.107188

**Published:** 2026-05-25

**Authors:** Ehab H. Mattar, Ali T. Zari, Esmail M. El-Fakharany, Yousra A. El-Maradny, Bassam O. Aljohny, Elrashdy M. Redwan

**Affiliations:** a Department of Biological Sciences, Faculty of Science, King Abdulaziz University, Jeddah, Saudi Arabia; b Princess Dr. Najla Bint Saud Al-Saud Center for Excellence Research in Biotechnology, King Abdulaziz University, Jeddah, Saudi Arabia; c Centre of Excellence in Bionanoscience Research, King Abdulaziz University, Jeddah 21589, Saudi Arabia; d Protein Research Department, Genetic Engineering and Biotechnology Research Institute (GEBRI), City of Scientific Research and Technological Applications (SRTA-City), 21934, New Borg AL-Arab, Alexandria, Egypt

**Keywords:** IgY nanocombination, Colistin-resistant bacteria, Antibacterial potential, Cytotoxicity, Salmonella

## Abstract

Egg yolk immunoglobulin Y (IgY) derived from avian sources, such as chickens, has attracted interest due to its low cost and rapid production. This study aimed to fabricate chitosan nanoparticles conjugated with IgY antibodies and polymyxin B as novel antibacterial nanocombinations to fight multi-resistant *Salmonella enterica* serovar Typhimurium (*Salmonella* Typhimurium) isolated from poultry. Chicken IgY was purified from egg yolk via caprylic acid precipitation and gel filtration. The developed polymyxin B-loaded nanocombinations, enhanced by IgY-polymyxin B-based NPs (IgY-Poly/ChNPs and IgY-coated-Poly/ChNPs), were designed to overcome the resistance limitations of polymyxin B. In this system, polymyxin B serves as the primary bactericidal agent, while the chitosan nanoparticle acts as a biocompatible carrier matrix, and the IgY coating serves as a functional shell to enhance stability and potentially interfere with bacterial surface attachment. The IgY-polymyxin B-based NPs exhibited a spherical morphology (124.2–290.7 nm) and a zeta potential of -26.5 to -46.9 mV. The formulations demonstrated potent inhibitory efficacy (12±0.98 – 21±0.91 mm) with MIC values of 0.125 to 2.0 mg/mL against *Salmonella* Typhimurium. These nanocombinations inhibited bacterial growth in a time- and dose-dependent manner. A significant downregulation of mcr-1 gene expression (3.97 to 8.7-fold) was observed in colistin-resistant *Salmonella* Typhimurium isolates, though this effect was isolate-dependent. SEM analysis showed extreme distortion in treated bacterial cells. Finally, the IgY-polymyxin B-based NPs showed minimal toxicity toward normal human skin fibroblast (HSF) cells, demonstrating them as possible candidates for future antimicrobial therapy.

## Introduction

The sole substance in the yolk that moves from the hen's serum and other birds to the egg yolk to provide passive protection to bird embryos and newly hatched birds is immunoglobulin Y (IgY), also known as egg yolk immunoglobulin (IgY antibody) (Radwan et al., 2023). Researchers are highly interested in IgY because these avian antibodies can be used as immunotherapeutic and immunodiagnostic candidates in a variety of life sciences fields and applications. In reality, exploring novel strategies to combat infection is essential in light of the growing threat of antibiotic resistance, and oral administration of manufactured particular antibodies is among the most alluring approaches available. Orally administered IgYs derived from chicken egg yolks can prevent and passively treat infectious illnesses of viral and bacterial source in both people and animals. Even though oral IgY treatment has several clear benefits, it can be difficult to extract pure IgY from egg yolk (Redwan et al., 2021). Because their high vitamin content, necessary fatty acid content, and huge amounts of amino acids, poultry eggs are a great source of energy, micronutrients, and protein content (Bohrer, 2017). Therefore, there are many attempts were carried out in the last decade to extract and harvest the IgY in a pure form for uses in the pharmaceutical industry. IgY is the sole immunoglobulin that translocates to egg yolk, which is easily recovered utilizing precipitation procedures (Redwan et al., 2021). Recently, IgY has attracted a lot of interest as an alternative for passive vaccination. Because chicken IgY does not activate mammalian complement components or bind to human Fc receptors, it is thought to be safer than other immunoglobulins like IgG and does not cause potentially harmful immunological reactions (Carlander et al., 1999). Chickens can produce eggs containing IgY immunoglobulins on a big scale and in significant quantities using non-humane and invasive approaches, potentially providing novel, cost-effective, and effective immunotherapy opportunities (Gadde et al., 2015). Due to the evolutionary difference between birds and mammals, IgY is produced against conserved proteins in mammals more easily and effectively than IgG in other human or mammals. IgY has a great ability to stimulate an effective immune response at low levels and to bind to target antigens compared to IgG in mammals (El-Kafrawy et al., 2023). It is currently estimated that 700,000 deaths per year are caused by antimicrobial resistance (AMR). By 2050, AMR may cause up to $100 trillion in global social costs, a 2% to 3.5% drop in GDP, and roughly 10 million deaths each year (O’Neill, 2019).

IgY has demonstrated efficacy against a variety of enteric pathogens, including rotavirus, Salmonella spp., and *Klebsiella pneumoniae*, by inhibiting their development and their symptoms both in vitro and in vivo models. As a result, studies have started to focus on encapsulating IgY to protect it from harsh conditions, aiming to preserve antibody activity against enzymatic degradation and pH destruction. At the same time, advances in nanotechnology are reshaping healthcare, with growing applications in diagnosis, treatment, and disease prevention. Progress in nanoscience has become particularly important in biomedicine, especially in areas such as drug delivery, medical devices, and therapeutic and diagnostic approaches, offering a potential natural option for future clinical use (Bayda et al., 2020). Interestingly, the reason these nanoscale materials work so well in drug delivery fields is because they have good biocompatibility and biodistribution characteristics. Fundamentally, nanoparticles as therapeutic agent carriers have the potential to significantly improve drug delivery because they can shield the loaded agents from deterioration, extending their stability and half-life (Malik et al., 2021). These features include enhanced antibacterial activity, barrier qualities, and sensing skills as well as lowering the chance of contamination and rise the shelf life of perishable goods (Gupta et al., 2024).

A variety of illness outbreaks in poultry are caused by bacteria, fungi, and viruses. For instance, salmonellosis in chicken is linked to non-typhoidal Salmonella serotypes. The zoonotic potential of salmonellosis might result from eating tainted meat and eggs. Contact with carrier animals, such as rats, cats, and insects, is one of the many ways that Salmonella can spread among chickens. Its transmission also involves contaminated water, waste, food supply, and aerosols (Shaji et al., 2023). Drugs used to treat infectious diseases are rendered less effective due to bacterial resistance. The World Health Organization (WHO) and several international scientific organizations concur that the rise in AMR necessitates the creation of a worldwide, a coordinated action plan to address it. The spread of resistance genes to more dangerous bacterial species and extended-spectrum beta-lactamase (ESBL) resistance in Salmonella and other Enterobacteriaceae is significant. The discovery of comparable molecular mechanisms for resistance in various strains lends support to the horizontal spread of resistance determinants. AMR microbes can survive and spread due to natural selection (Aslam et al., 2018).

In addition, colistin-resistant bacteria (CRB) is a rising public health concern, emerging from widespread uses of antibiotic in agriculture and poultry fields, where mobile colistin resistance (mcr) genes on plasmids provide rapid spread, often alongside other resistances. These resistant strains can transfer from infected animals to humans via the food chain, posing a threat because colistin is a last-resort treatment for severe multidrug-resistant infections after other treatments have limited (Lima et al., 2019). Due to the limited or nonexistent effective treatment options, colistin-resistant bacteria can lead to increased morbidity and mortality rates. Individuals exposed to colistin-resistant microbes, particularly Salmonella, are more likely to experience treatment failure, prolonged illness, and worsening health conditions. Therefore, this study aims to develop a novel nanocarrier system for the co-delivery of egg yolk IgY and polymyxin B using a chitosan nanoplatform to address the multi-resistance challenge of pathogens such as *Salmonella* Typhimurium. In this system, polymyxin B serves as the primary antimicrobial agent, while the chitosan-IgY matrix is designed to protect the payload from enzyme-mediated hydrolysis, enhance its interaction with the bacterial surface, reducing side effects, and lowering the dose through high encapsulation in a single dose.

## Methodology

### Processing of eggs Yolk

Fresh white-shelled chicken eggs were obtained from a local market and kept refrigerated until yolk extraction. The eggs were carefully opened by hand, and most of the egg white (albumin) was removed by rinsing with cold water. The yolk was then gently transferred onto filter paper to eliminate any remaining albumin attached to the yolk membrane. After that, the membrane was punctured, and the yolk was collected into a glass beaker placed in an ice bath. Following gentle mixing for about 10 minutes, the yolk was diluted with cold water, and the pH was adjusted to around 4.9–5.0. The resulting sample was gently mixed, left at room temperature for 2 hours and centrifuged for 30 minutes at 3800 rpm in cold condition. Then, the supernatant samples were pooled, while the precipitates were discarded.

### Purification of chicken IgY from eggs yolk

Chicken IgY was purified by precipitation method following the protocol of Redwan et al. (Redwan et al., 2021). Briefly, three stages of gradual addition of 6% caprylic acid (CA) to the filtrate were used to isolate and purify IgY. In the first step, 2% CA was added dropwise to the filtrate with gentle stirring (350–750 rpm), while maintaining the pH at 5.0. The sample was then stirred for 30–35 minutes at room temperature and centrifuged at 3800 rpm for 30 minutes at 4°C. After carefully removing the disc formed at the top of the sample, the precipitate was discarded, and the filtrate was collected. The filtrate was subjected to the same treatment for a second round with an additional 2% CA. A third 2% CA was then added, and the entire procedure was repeated. After the total addition of 6% CA in three batches (2%, 2%, and 2%), the final filtrate was ultrafiltered to concentrate the IgY sample, remove low-molecular-weight components, and exchange the buffer to 50 mM Tris-HCl (pH 8.0) using Amicon Ultra-15 centrifugal filter units. The IgY contained sample was applied into a pre-equilibrated Sephacryl-S200 size exclusion column (5 × 150 mm, GE Healthcare, Sweden) with a Tris-HCl buffer solution (pH 8.0) containing 0.15 mM sodium chloride. Proteins were subsequently eluted using an AKTAprime Plus FPLC system (GE Healthcare, Cytiva) with the same buffer.

### Protein‐chemistry characterization

• Homogeneity and purity of IgY was estimated by 12% SDS-PAGE and Coomassie brilliant blue was used to stain protein gels.
• The concentration of IgY in the samples was determined based on the Bradford method (Bradford, 1976), following the producer manual's microplate assay technique using bovine serum albumin (BSA) to perform the standard curve. (y = 0.0004x + 0.0054)
• For western blot analysis, purified IgY samples were resolved on 12% SDS–PAGE gels and then transferred onto a nitrocellulose membrane using a transfer buffer (pH 8.4) composed of 1.93 g/L Tris base and 9 g/L glycine. The transfer was carried out at 30 V for 8 h. The membrane was then blocked with 2% (w/v) BSA in PBS for 1 h. After washing with PBS, it was incubated with alkaline phosphatase–conjugated AffiniPure rabbit anti-chicken IgY (#303-055-003; Jackson ImmunoResearch Laboratories, Inc., West Grove, PA) for 1 h at 37°C. After washing, protein bands are developed with bromochloroindolyl phosphate/nitro blue tetrazolium (BCIP/NBT).

### Preparation of IgY nanocarriers

Chitosan nanoparticles (ChNPs) was designed and fabricated through using the ionic gelation process, following the protocol of Anitha et al. (Anitha et al., 2011), with a few changes. Low molecular weight chitosan (2.0 mg/mL) was dissolved in 0.1% glacial acetic acid through continuous gentle stirring until complete dissolving and pH was adjusted to 5.5. The dissolved IgY (0.5 mg/mL) in 50 mM phosphate buffer pH, 7.2 and dissolved polymyxin B (2 mg/mL) in deionized water, both solutions or mixture of IgY and polymyxin B were separately added dropwise to the chitosan solution over 1 hour. A dextran sulfate (0.5 g/L) was prepared in deionized water and used as a cross-linking agent. IgY- and polymyxin B loaded chitosan nanoparticles were formed spontaneously by slowly adding an equal volume of dextran sulfate solution under continuous stirring at 800 rpm overnight. Following the formation of IgY-loaded (IgY/ChNPs), polymyxin-loaded (Poly/ChNPs), and co-loaded (IgY-Poly/ChNPs) nanoparticles, the encapsulation efficiency of both IgY and polymyxin B was calculated using the following equation: Encaps %= [(A-B) /A] × 100, where (A) is the total concentration of IgY or polymyxin B and (B) is the free content of IgY or polymyxin B. Then, the mixture of Poly/ChNPs was divided into two parts and centrifuged at 12000 rpm in cold condition for 30 min to obtain uncoated NPs.

### Surface coating with IgY

**Fig. 1:**
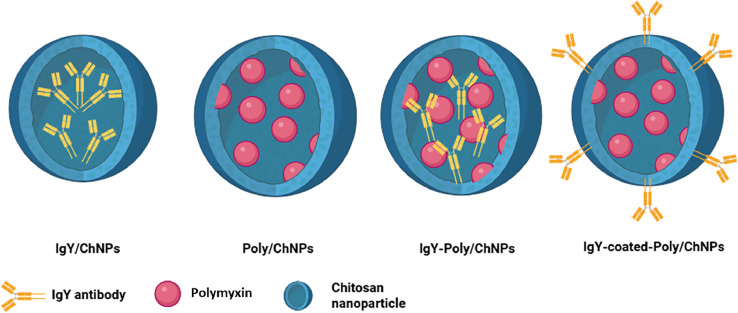
Schematic illustration of the different nanocombination formulations.

For coating, the purified IgY (5 mg/mL) was added dropwise over 60 min to one part of Poly/ChNPs resuspended in 0.05 M Tris-HCl buffer, pH 9.0. After centrifugation at 12000 rpm for 30 min at 4°C, the developed IgY-coated-Poly/ChNPs (Fig. 1) was redissolved in 1x PBS, pH 7.2 and freeze-dried for further uses. Unencapsulated drugs and excess coating polymers was removed by centrifugation and dialysis methods.

### Physicochemical characterization of nanocarriers

The developed formulations were evaluated for key physicochemical properties. Particle size and polydispersity index (PDI) were determined using dynamic light scattering (DLS). Surface charge (zeta potential) was measured by electrophoretic light scattering to assess stability and confirm surface characteristics. The morphology of the synthesized nanoparticles was examined using scanning electron microscopy (SEM) at an accelerating voltage of 15 kV.

### Cytotoxicity assessment

The cytotoxic effect of the IgY-polymyxin B-based NPs was assessed using the MTT assay. Normal human skin fibroblast (HSF) cells were seeded in 96-well plates at a density of 1 × 10^3^ cells per well and allowed to attach overnight. Cells were then exposed to different nanoformulations at concentrations ranging from 0 to 2 mg/mL for IgY-polymyxin B-based NPs and 0 to 0.2 mg/mL for polymyxin B, followed by 48 h incubation. Formazan formation was quantified by measuring absorbance at 570 nm, and values of EC_50_ were calculated via GraphPad Prism (version 9.0).

### Isolation of *Salmonella enterica* serovar typhimurium from ready-to-cook chicken

A total of 50 samples from raw chicken meat (ready-to-cook) intended for human consumption were collected and screened for bacterial isolation following previously described procedures (Ashrafudoulla et al., 2021). Briefly, 25 g of chicken muscle tissue from each sample were sterilely transferred into sterile bags containing 225 ml of 2% diluted peptone water and homogenized for 2 minutes under sterile conditions. Samples were incubated at 37°C for 24 h for pre-enrichment. Subsequently, 100 µL of each culture was inoculated into Rappaport–Vassiliadis (RV) broth and cultured for 24 hours at 42°C for selective enrichment. Enriched cultures were streaked onto selective agar plates such as Xylose Lysine Deoxycholate [XLD] agar and incubated for 24 h at 37°C. Suspected colonies were selected based on morphology and subcultured to obtain pure isolates.

### Identification and antimicrobial susceptibility

Presumptive isolates were identified utilizing the VITEK® 2 automated system (bioMérieux, France) with Gram-negative identification cards. Profiling of antimicrobial susceptibility testing was carried out using the corresponding VITEK® 2 AST panels according to the manufacturer’s instructions. The multidrug resistance was identified based on CLSI criteria as resistance to at least one candidate in three or more antimicrobial classes (CLSI M100, 2026).

### Screening for colistin resistance

Colistin susceptibility was evaluated using the broth microdilution method following EUCAST and CLSI guidelines. A stock solution of polymyxin B (1 mg/mL) was sterilized by filtration (0.22 µm), and serial two-fold dilutions were prepared in cation-adjusted Mueller–Hinton broth to achieve concentrations ranging from 0.39 to 200 µg/mL. Microplates were inoculated with 20 µL of overnight cultures adjusted to approximately 10⁵ CFU/mL. *E. coli* ATCC 25922 was included as a quality control strain. After incubation at 37°C for 24 h, MIC values were recorded in triplicate. Isolates with MIC values above 2 µg/mL were considered resistant according to EUCAST criteria (EUCAST, 2021).

## Evaluation of antibacterial activity of IgY formulations

### Agar well diffusion assay

Antibacterial activity was assessed using the agar well diffusion approach. Mueller–Hinton agar plates were inoculated with colistin-non-susceptible *S. enterica* serovar Typhimurium adjusted to a 0.5 McFarland standard. 8 mm wells were prepared, and 50 µL of each formulation was added. Plates were cultured at 35–37°C for overnight, and inhibition zones were estimated in triplicate.

### Determination of MICs of IgY formulations

Values of MIC were determined using the broth microdilution method in 96-well plates. Briefly, IgY-polymyxin B-based NPs were serially diluted in Mueller–Hinton broth (MHB). Wells were inoculated with 50 µL of an 18-hour bacterial culture adjusted to 0.5 McFarland standard. Negative control wells contained MHB supplemented with the corresponding concentrations of IgY formulations without bacterial inoculation. Positive control wells contained bacterial inoculum in MHB without IgY formulations. Sterility control wells contained uninoculated MHB only. The microplates were cultured at 37°C for 18–24 hours under aerobic conditions. The MIC was identified as the lowest dose of the IgY-polymyxin B-based NPs that showing clear well without turbidity, indicating inhibition of bacterial growth.

### Time–kill kinetic assay

Time–kill kinetics were evaluated in MHB supplemented with IgY-polymyxin B-based NPs at ½ MIC and MIC doses. Each flask was inoculated with exponentially growing *S. enterica* serovar Typhimurium at an initial density of approximately 10^5^ CFU/mL and cultured at 37°C in a shaking incubator (150 rpm). At periods of 0, 1, 6, 12, 18, and 24 h, aliquots were collected in triplicate from each treatment, along with a positive growth control consisting of inoculated medium without IgY. Samples were diluted ten times in sterile 0.9% (w/v) saline, and 100 µL of each dilution was spread onto nutrient agar plates. After which plates were incubated at 37°C for 24 h, viable counts were determined and expressed as CFU/mL. The time–kill curves were assembled by plotting changes in bacterial counts over time. A bactericidal effect was defined as a ≥ 3 log_10_ decrease in CFU/mL at 24 h relative to the initial inoculum, whereas reductions <3 log_10_ were interpreted as bacteriostatic activity (El-Sherbiny et al., 2024).

CFU/mL=(Numberofcolonies×Dilutionfactor)/Volumeplated(mL).

### Antibiofilm activity assay

Biofilm formation and disruption were evaluated using a crystal violet staining assay on an abiotic surface, with minor adaptations from previously established protocols. Briefly, sterile 96-well tissue culture–treated polystyrene microplates were prepared with 100 µL of tryptic soy broth (TSB) supplemented with 2% glucose then adding the IgY-polymyxin B-based NPs at the MIC concentrations. Wells were then inoculated with colistin-non-susceptible *S. enterica* serovar Typhimurium isolates suspensions prepared from overnight cultures and adjusted to a standardized density equivalent to a 0.5 McFarland standard, followed by appropriate dilution in fresh medium. Plates were incubated at 37°C for 48 h under static conditions to allow biofilm development. The culture medium was carefully removed, and wells were gently washed twice with sterile PBS to remove non-adherent bacterial cells. The remaining attached biofilms were air-dried and stained with 0.1% (w/v) crystal violet solution at room condition for 15 min. The wells were washed to discard excess stain through rinsing three times in PBS and the bound dye was air dried then solubilized utilizing an acetone: ethanol mixture (30:70, v/v). Biofilm biomass was calculated by measuring OD at 570 nm using a microplate reader. Wells containing medium alone were included as negative controls, while untreated bacterial cultures served as positive biofilm controls (Felipe et al., 2019).

For the evaluation of biofilm eradication, biofilms were first allowed to establish for 24 h under the conditions described above. The planktonic phase was then removed, and fresh medium containing IgY-polymyxin B-based NPs was added to the preformed biofilms. Plates were cultured at 37°C for an additional 24 h and residual biofilm biomass was subsequently determined utilizing the crystal violet staining described above (Ahmed et al., 2025). The percentage of biofilm inhibition was considered relative to the untreated control. All experiments were tested in triplicate, and findings were stated as mean ± standard deviation (SD).

BiofilmInhibition(%)=((ODcontrol−ODsample)/ODcontrol)×100

%Ofbiofilm=100−BiofilmInhibition(∖%)

### Expression of resistant genes in salmonella following IgY formulations treatment

Colistin-non-susceptible *Salmonella* Typhimurium isolates were exposed to sub-inhibitory concentrations of IgY-polymyxin B-based NPs. Total bacterial RNA was extracted utilizing the EasyPure RNA Mini Kit (TransGen Biotech, China) and quantified using a Qubit® 3.0 fluorometer. Complementary DNA was synthesized from 400 ng RNA utilizing the QuantiTect Reverse Transcription Kit (Qiagen, Germany). Real-time qPCR was performed using SYBR Green chemistry using primers in Table 1. Conditions of cDNA amplification contained of an initial denaturation step at 95°C for 10 min, followed by 40 cycles step at 95°C for 15 s, 60°C for 40 s, and 72°C for 30 s. Relative gene expression was estimated utilizing the ΔΔCt method with the 16S rRNA gene as the internal reference. Statistical significance was determined using Student’s *t*-test (P < 0.05).

**Table 1:** Primers used for the identification of mcr-1–mediated colistin resistance.

Target gene	Primer name	Sequence (5′–3′)	Application	Reference
mcr-1	mcr-1-F	GGCGTATTCTGTGCCGTGTA	RT-qPCR	(Liu et al., 2016)
	mcr-1-R	CGTGATCGCGTCATGGGT	RT-qPCR	(Liu et al., 2016)
mcr-1 (variant)	M1-F	ACACTTATGGCACGGTCTATG	RT-qPCR	(Cimmino et al., 2016)
	M1-R	GCACACCCAAACCAATGATAC	RT-qPCR	(Cimmino et al., 2016)
16S rRNA	27-F	AGAGTTTGATYMTGGCTCAG	Housekeeping gene	(Kim et al., 2012)
	515-R	TTACCGCGGCKGCTGGCAC	Housekeeping gene	(Kim et al., 2012)

### Scanning electron microscopy

Two representative colistin-non-susceptible isolates were cultured in CA-MHB supplemented with IgY-polymyxin B-based NPs at MIC and ½ MIC concentrations. Bacterial cultures were incubated at 37°C for 18-24 hours with shaking. Bacterial cells were harvested, processed, and examined using a JEOL JEM-1400 scanning electron microscope following standard protocols.

### Statistical analysis

All experiments were achieved in triplicates and data were expressed as mean ± standard deviation. Statistical analysis was conducted via GraphPad Prism 9.0. The differences among groups were evaluated using two-way ANOVA, with P < 0.05 reflected statistically significant.

## Results and discussion

### Purification of IgY antibodies

**Fig. 2:**
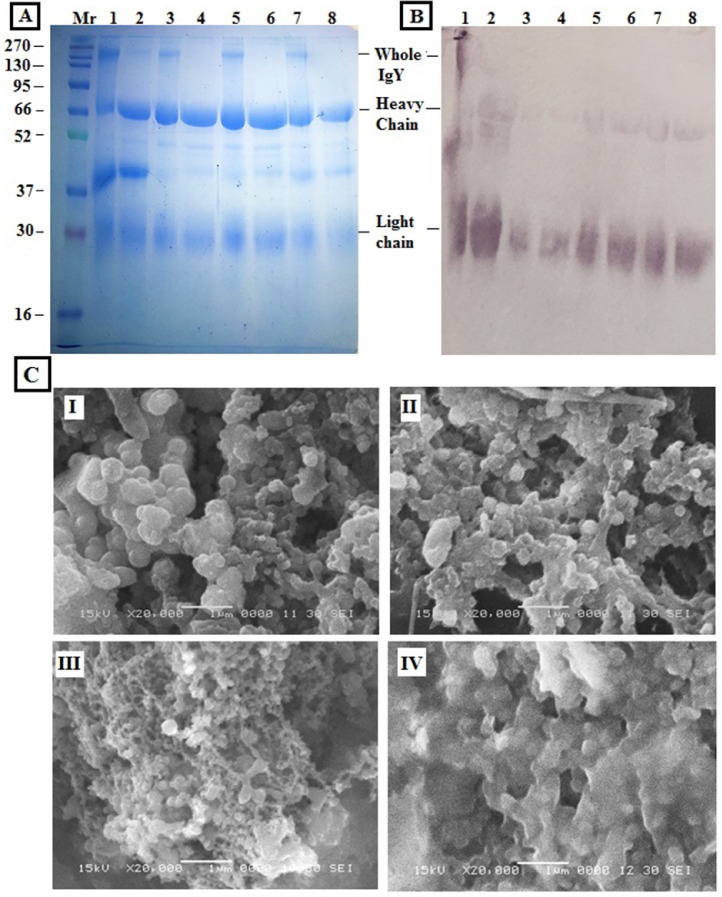
The purification steps of chicken IgY under non-reducing and reducing conditions, as confirmed by (**A**) 12% SDS-PAGE and (**B**) western blot analysis. Lane Mr is protein ladder, lanes 1 and 2 are the precipitated IgY using serial 2 + 2 + 2% addition of caprylic acid under non-reducing and reducing conditions, respectively, lanes 3, 5, 7 are the purified IgY fractions using Sephacryl S200 column under non-reducing condition, lanes 4, 6, 8 are the purified IgY fractions using Sephacryl S200 column under reducing condition, (**C**) SEM imaging of the prepared IgY NPs (I) IgY/ChNPs, (II) Poly/ChNPs, (III) IgY-Poly/ChNPs and (IV) IgYcoated-Poly/ChNPs.

Recently, many nanomaterials have been developed very rapidly for use in a wide range of uses in numerous medical fields, comprising targeted drug delivery systems, scaffolds and tissue engineering devices, cancer treatment, diagnostics, and clinical analytical biotherapies (Bakhshi et al., 2017). Due to the biocompatibility and biodegradability of chitosan nanoparticles, there has been increasing interest in their ability to act as an effective carrier for delivering vaccines, drugs, genes, and other biomolecules to specific sites in the body. However, some harsh conditions such as low pH and the presence of different types of proteases makes the use of chitosan nanoparticles suitable, particularly in the case of delivering protein-based drugs (Katas et al., 2013). In this study, we used the CA method to purify IgY from chicken egg yolks. This method is a classic purification procedure, low-cost, simple, and appropriate for large-scale production (Redwan et al., 2021). After diluting the egg yolks with cold water at ratio of 1:5, a solution of CA at concentration of 2 + 2 + 2% at pH 5.0 was provided to the diluted egg yolk in a separate step. The obtained findings indicated that the serial addition of CA (2 + 2 + 2%) at pH 5.0 is suitable for removing non-IgY proteins. Fig. 2A shows the results of the purification steps of chicken IgY using serial addition of CA (2 + 2 + 2%), which indicating the disappearance of non-IgY proteins from the supernatants solution with appearance of band at 43 kDa that corresponding to egg albumin (lanes 1 and 2). After a further purification step using the gel filtration method, this band disappeared or appeared slightly in the eluted fractions from the Sephacryl-S200 column (lanes 3-8). The results obtained under non-reduced condition exhibited that IgY involved more than 1 band at 180 kDa for whole IgY, and 65 and 27 kDa for heavy and light chains, respectively. However, the results under reduced conditions exhibited that β-mercaptoethanol reduced the whole IgY band to give two main bands at 65 kDa and 27 kDa for heavy and light chains (Fig. 2A). Rabbit IgG anti-chicken IgY antibody labelled alkaline phosphatase-antibody was used to identify the purified IgY and two chains using the western blot approach. Fig. 2B showed two bands for both heavy and light chains at 65 and 27 kDa, which confirmed that the purified protein from the egg yolk was the chicken IgY.

### Preparation and characterization of IgY-polymyxin B nanocombinations

The encapsulation of IgY in chitosan NPs (IgY/ChNPs) demonstrated high efficiency (86%), while polymyxin B-loaded NPs (Poly/ChNPs) achieved an encapsulation efficiency of 73%. During the coating process, the coating efficiency of IgY on Poly/ChNPs was determined to be approximately 81%. Nano-size analysis of all fabricated nanocombinations indicated that the size of IgY/ChNPs, Poly/ChNPs, IgY-Poly/ChNPs and IgY-coated-Poly/ChNPs estimated to be ranged from 124.2 to 290.7 nm (Table 2). In addition, zeta potentials of IgY/ChNPs, Poly/ChNPs, IgY-Poly/ChNPs and IgY-coated-Poly/ChNPs were −28.7, −28.3, −46.9 and −26.5 mV, respectively (Table 2). IgY, polymyxin B or IgY+polymyxin B loaded chitosan–dextran sulphate NPs were synthesized directly owing to the co-acervation reaction between both negatively charged dextran sulphate and positively charged chitosan. The co-acervation reaction occurs due to the electrostatic contact between the sulfate groups in dextran sulfate and the protonated amino groups in chitosan. The surface charge and size of the formed NPs can be modified by changing the reaction conditions and the polymer concentration, which make these NPs exhibited a suitable stability and do not necessitate any stabilizing agents (Anitha et al, 2011). There were several studies revealed that the nanocombination of chitosan–dextran sulphate NPs acts as an efficient and versatile, controlled-release drug delivery system, particularly leveraging their ability to form stable, oppositely charged polyelectrolyte complexes. They also have been reported for use as a controlled drug delivery for cancer therapy, oral delivery of insulin and intravenous delivery of anti-angiogenesis peptides (Chen et al., 2003; Fathy et al., 2023). The negative surface charge of the fabricated IgY, polymyxin B or IgY+polymyxin B loaded chitosan-dextran sulphate NPs may be attributed to the presence of un-neutralized or excess dextran sulphate segments on the outer shell of the formed particles. This finding is matched with the findings of Guarino et al., (Guarino et al., 2021) who demonstrated that negatively charged of chitosan-dextran sulfate NPs contain dextran sulfate in their outer shell, which is useful for delivering and integrating of therapeutic heparin-binding proteins. Results from zeta size and potentials showed that the coating of IgY slightly decreased the surface potential of Poly/ChNPs and increased their size. Negatively charged NPs or those with neutral zeta potential often demonstrate a better transported and cellular uptake via Peyer's patches compared to positively charged NPs (He et al., 2010).The morphology of the fabricated IgY/ChNPs, Poly/ChNPs, IgY-Poly/ChNPs and IgY-coated-Poly/ChNPs demonstrated a spherical or semi-spherical shapes as shown in Fig. 2C.

**Table 2:** Physicochemical characteristics of the prepared nanocombinations, including particle nanosize, zeta potential (surface charge), and polydispersity index (PdI).

Compounds	Zeta size (nm)	Zeta potential (mV)	PdI
IgY/ChNPs	165.9 ± 15.95	−28.7 ± 4.31	0.319
Poly/ChNPs	124.2 ± 18.09	−28.3 ± 4.34	0.384
IgY-Poly/ChNPs	198.1 ± 15.11	−46.9 ± 4.46	0.536
IgY-coated-Poly/ChNPs	290.7 ± 18.59	−26.5 ± 7.54	0.412

### FTIR spectroscopy assessment of IgY-polymyxin B nanocombinations

**Fig. 3:**
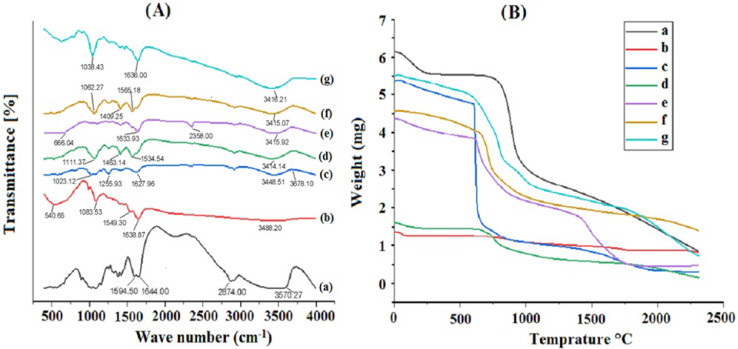
FTIR Spectra (**A**) and TGA thermograph curves (**B**) of chitosan (a), IgY (b), IgY/ChNPs (c), polymyxin B (d), poly/ChNPs (e), IgY-Poly/ChNPs (f) and IgY-coated- Poly/ChNPs (g).

FTIR analysis is an operative fingerprinting approach that identifies substances dependent on their unique chemical structures. Fig. 3A depicts the FTIR analysis of chitosan, chicken IgY, Poly b, IgY/ChNPs, Poly/ChNPs, IgY-Poly/ChNPs and IgY-coated-Poly/ChNPs. The FTIR spectra of the purified IgY displayed distinct typical absorption bands in the amide I region (1600–1700 cm^-1^) and amide III region (1220–1330 cm^-1^), which are particularly informative for investigating the secondary structure of proteins (Miller et al., 2013). The purified IgY and IgY-coated-Poly/ChNPs depicted slight differences. Alterations of amide I and amide II bands are influenced by electrostatic interactions between proteins and other surrounding molecules. For IgY-coated-Poly/ChNPs spectra, a slight modification at 1636.00 cm^-1^ and 1038.43 cm^-1^. The characteristic absorption of O—H from 3570.27 cm^−1^ for chitosan to be around 3414-3448 cm^−1^ was detected after for all fabricated IIgY-polymyxin B-based NPs, which confirmed the formation of stronger intermolecular and intramolecular hydrogen bonds between the protein and chitosan. Also, the characteristic absorption of C—O-C and C—O stretching in chitosan from 1152.61 to be around 1038-1111 cm^−1^ enhanced with the incorporation of IgY with chitosan, which often enhanced by hydrogen bonding between the amine/hydroxyl groups of chitosan and the protein residues in IgY. The bands at 1627-1636 cm^-1^ in all fabricated NPs depicted C = O stretching of amide I vibrations, which are often associated with β-sheet conformations within the protein structure coating the nanoparticles (Sadat and Joye, 2020). The band at 1565.18 cm^-1^ in IgY-Poly/ChNPs associated to the N—H bending vibration of the amide II band as a key indicator of residual N-acetyl groups (Lim and Hudson, 2004). The vibrational bands located outside the plane of the monosaccharide ring in the fabricated NPs were observed at approximately 1400–1460 cm⁻¹ (CH₂ bending), 1025–1062 cm⁻¹ (C–O stretching), and 795–895 cm⁻¹ (C–H bending) (Queiroz et al., 2015). FTIR analysis of polymyxin B shows characteristic peaks around 3300-3414 attributed to O—H and N—H stretching (Amide I and Amide II). The spectra reveal amide and amino groups indicative of its cyclic peptide structure and fatty acyl tail. Additionally, the bands that appeared for the polymyxin B spectrum at 1650.35 cm^-1^ (C = O stretching), 1534.54 (N—H bending) of amide II, and the bands at 2927 to 2955 cm^-1^ were attributable to C—H stretching from the fatty acid moiety (Gulani et al., 2025). These peaks were shifted along with presence of 1633.94, 1411.05, and 2848.2 cm^-1^ bands, which appeared in Poly/ChNPs. Consequently, the encapsulation of polymyxin B into the network of chitosan NPs was evidenced by the changed FTIR spectra, which indicate chemical interactions (Yousfan et al., 2024). However, the bands at 1636.00 and 1038.43 cm⁻¹ were more intense in the IgY-coated Poly/ChNPs (Fig. 3A), which may indicate the formation of hydrogen bonds. These interactions likely arise from the presence of multiple functional groups, including –NH₂ groups in IgY and chitosan, –OH groups in both IgY and chitosan, and –COOH groups in the protein structure (Tavassoli et al., 2021).

### Thermostabilty of IgY-polymyxin B nanocombinations assessment

To further assess the structural stability, TGA was performed to evaluate the effect of the fabricated IgY-polymyxin B-based NPs on the thermal degradation behavior of purified IgY, as shown in Fig. 3B. It was revealed that the 1st degradation phase in the graph displayed a higher moisture absorption in all fabricated IgY/ChNPs, Poly/ChNPs, IgY-Poly/ChNPs and IgY-coated-Poly/ChNPs due to the elimination of moisture in the fabricated NPs (≤ 100°C). The 2^nd^ degradation phase of graphs proves that the temperature of weight loss has increased progressively for IgY/ChNPs, Poly/ChNPs, IgY-Poly/ChNPs and IgY-coated-Poly/ChNPs (224 to 254°C, 236 to 284°C, 234 to 344°C and 202 to 272°C, respectively). TGA analysis profile revealed the thermal decomposition of IgY/ChNPs (approximately 80%) at a low temperature between 43.03 and 438.50°C, whereas the fabricated Poly/ChNPs exhibited thermal decomposition (around 60%) at 34 and 479°C. The encapsulation of both IgY and polymyxin B into chitosan NPs (IgY-Poly/ChNPs) showed an increase in the thermal decompose than IgY-coated form (IgY-coated-Poly/ChNPs). As observed, the residues were also increased to the thermal decomposition in all fabricated NPs containing polymyxin B in the 3rd stage than the fabricated IgY/ChNPs, which provides higher thermally stable pattern. The zeta potential (surface charge) of the chitosan shifted from positive to negative following the incorporation of negatively charged dextran sulfate. This change reflects surface charge reversal and contributes to improved dispersion stability by increasing the magnitude of the particle’s net surface charge. During nanoparticle formation, electrostatic complexation occurs between positively charged chitosan and negatively charged dextran sulfate, and when dextran sulfate predominates at the particle surface, the zeta potential shifts to negative values. This charge reversal has been widely reported in chitosan–dextran sulfate polyelectrolyte complexes and is considered evidence of successful coating or surface localization of dextran sulfate (Chen et al., 2003; Guarino et al., 2021).

### In vitro cytotoxicity assessment on normal cell lines

**Fig. 4:**
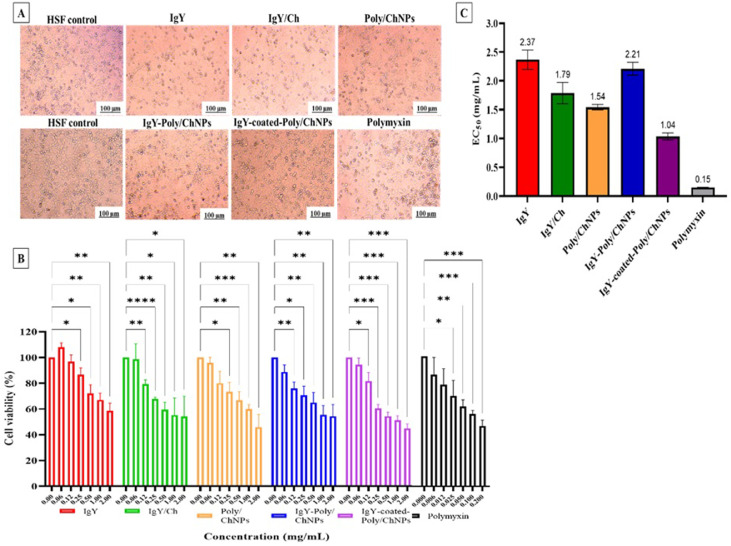
(**A**) Treatment-induced morphological alterations in normal human fibroblast (HSF) by inverted phase-contrast microscopy. **(B)** The cytotoxic effect of the prepared nanocombinations on the growth of HSF cell line was evaluated *in vitro* following 48 h of exposure using the MTT assay. **(C)** EC_50_ values of the tested compounds in HSF cells were determined using nonlinear regression analysis of normalized response data. Data are presented as mean ± SD. Statistical significance relative to the untreated control is indicated as *P < 0.05, **P < 0.01, ***P < 0.001, and ****P < 0.0001.

The cytotoxic profile of the tested formulations was evaluated *in vitro* utilizing HSF normal cell lines, and cell viability was quantified across a range of added concentrations. As illustrated in Fig. 4, all treatments exhibited a clear concentration-dependent reduction in cell viability. The calculated EC₅₀ values indicated a generally acceptable safety margin for the investigated formulations. Among the tested groups, free IgY exhibited the highest EC_50_ value (2.37 mg/mL), followed by IgY/ChNPs (1.79 mg/mL) and IgY-Poly/ChNPs (2.21 mg/mL). A further reduction in EC₅₀ was observed for Poly/ChNPs (1.54 mg/mL), whereas IgY-coated-Poly/ChNPs showed a more pronounced cytotoxic effect with an EC_50_ of 1.04 mg/mL. Polymyxin B demonstrated the lowest EC_50_ value (0.15 mg/mL), reflecting its comparatively higher cytotoxicity toward normal cells. Phase-contrast microscopic examination further supported the quantitative viability data. Untreated control cells exhibited normal morphology, characterized by intact cell membranes, typical spindle or polygonal shapes, and firm attachment to the culture surface. At higher treatment concentrations, cells showed morphological alterations, including cell rounding, reduced adherence, shrinkage, and irregular outlines, indicating stress-related cytotoxic effects. In contrast, cells exposed to lower concentrations retained their normal morphology, with no obvious signs of membrane damage or detachment, comparable to control cultures.

### Isolation of colistin-resistant bacteria

The increasing occurrence of colistin-resistant strains has prompted efforts to identify alternative therapies for bacterial infections caused by multidrug-resistant Gram-negative bacteria. A high prevalence of resistance has been documented in animal populations, whereas reported rates in humans are generally lower, typically below 10%, with most cases described in Mediterranean countries and Southeast Asia (Huang et al., 2017). These observations suggest that animal reservoirs may contribute to the emergence and transmission of colistin resistance in human populations. Five Gram-negative bacterial isolates were recovered from raw chicken (poultry) samples using MacConkey agar. In addition, *Escherichia coli* ATCC 25922 was included as a quality control reference strain for colistin susceptibility assay. The minimum inhibitory concentrations (MICs), presented in Table 3, of colistin were estimated for all poultry-derived isolates using the broth microdilution method, in accordance with EUCAST recommendations (EUCAST, 2019), which recognize this method as the reference standard for colistin MIC determination. Based on the EUCAST colistin breakpoint (≤ 2 µg/mL), one isolate exhibited an MIC of ≤ 0.25 µg/mL and was classified as colistin-susceptible. In contrast, three isolates showed elevated MIC values of 0.5 µg/mL, 0.5 µg/mL and 4.0 µg/mL, which classified as colistin-intermediate and colistin-resistant. Overall, two out of five poultry-derived isolates (40%) were intermediate to colistin, while two out of five (40%) was classified as resistant. The quality control *E. coli* ATCC 25922 strain yielded an MIC of ≤ 0.25 µg/mL, which fell within the accepted EUCAST quality control range, thereby confirming the reliability of the susceptibility testing.

**Table 3:** Isolates MIC values of colistin.

Isolates	MIC value (µg/mL)	Isolates	MIC value (µg/mL)
**PS 1**	0.5	**PK 1**	8
**PS 2**	0.5	**PS 4**	≤ 0.25
**PS 3**	4.0	***E coli* ATCC 25922**	≤ 0.25

### Identification and antibiotic susceptibility profiling of colistin-resistant bacteria

The isolates recovered from raw chicken samples were identified utilizing the VITEK® 2 automated definition system. The isolates were identified as *Klebsiella pneumonia* and *Salmonella* Typhimurium. One isolate was identified as *K. pneumonia*, while the remaining four isolates belonged to *Salmonella* Typhimurium. The phenotypic antimicrobial susceptibility profiles of the colistin-intermediate and resistant isolates are summarized in Table 4. The results showed distinct resistance patterns among the tested poultry-derived isolates. All isolates exhibited resistance to aminoglycosides, as evidenced by uniform resistance to amikacin, gentamicin, and tobramycin. In addition, variability in resistance to β-lactam antibiotics was observed across the isolates. Isolates PS 1 and PS 2 demonstrated overall similar susceptibility profiles, remaining susceptible to most β-lactams, including ampicillin/sulbactam, piperacillin, piperacillin/tazobactam, ceftazidime, aztreonam, and carbapenems. However, both isolates showed intermediate susceptibility to ciprofloxacin and levofloxacin, in addition to intermediate susceptibility to colistin (MIC = 0.5 µg/mL). Despite this, resistance to multiple antimicrobial classes was evident due to their consistent resistance to aminoglycosides. In contrast, isolate PS 3 displayed a markedly broader resistance profile. This isolate was resistant to piperacillin, ceftazidime, meropenem, aminoglycosides, and colistin (MIC = 4 µg/mL), while remaining susceptible to carbapenems such as imipenem, fluoroquinolones, tigecycline, minocycline, and trimethoprim/sulfamethoxazole. The resistance to multiple β-lactams and aminoglycosides highlights a more concerning phenotype in this isolate. Based on the observed susceptibility patterns, all three colistin-non-susceptible isolates fulfilled the criteria for multidrug resistance, identified as resistance to at least one anti-bacterial candidate in three or more distinct anti-bacterial classes, according to CLSI guidelines.

**Table 4:** Phenotypic antimicrobial susceptibility of colistin-intermediate and resistant bacterial isolates utilizing the VITEK® 2 automated system. *= Advanced Expert System (AES) modified.

Antimicrobial	PS 1		PS 2		PS 3	
	MIC (µg/mL)	Interpretation	MIC (µg/mL)	Interpretation	MIC (µg/mL)	Interpretation
Ampicillin/Sulbactam	≤ 2	S	≤ 2	S	≤ 2	S
Piperacillin	≤ 4	S	≤ 4	S	≥ 128	R
Piperacillin/Tazobactam	≤ 4	S	≤ 4	S	≤ 4	S
Ceftazidime	0.25	S	0.5	S	≥ 64	R
Aztreonam	≤ 1	S	≤ 1	S	≤ 1	S
Imipenem	≤ 0.25	S	≤ 0.25	S	0.5	S
Meropenem	0.5	S	≤ 0.25	S	≥ 16	R
Amikacin	≤ 1	R*	≤ 1	R*	≤ 1	R*
Gentamicin	≤ 1	R*	≤ 1	R*	≤ 1	R*
Tobramycin	≤ 1	R*	≤ 1	R*	≤ 1	R*
Ciprofloxacin	0.5	I	0.5	I	≤ 0.06	S
Levofloxacin	0.5	I	0.5	I	≤ 0.12	S
Minocycline	≤ 0.5	S	≤ 0.5	S	2	S
Tigecycline	≤ 0.5	S	≤ 0.5	S	≤ 0.5	S
Colistin	0.5	I	0.5	I	4	R
Trimethoprim/Sulfamethoxazole	≤ 20	S	≤ 20	S	≤ 20	S

### =Agar well diffusion assay and determination of minimum inhibitory concentrations (MICs)

The antibacterial activity of the tested formulations against colistin-non-susceptible *Salmonella* Typhimurium isolates was further quantified by determining their MICs, as summarized in Table 5. Clear differences in antibacterial efficacy were observed among the tested compounds. Neither free IgY nor IgY/ChNPs produced measurable inhibition zones or detectable MIC values within the tested concentration range, indicating a lack of direct antibacterial activity against all isolates. In contrast, Poly/ChNPs exhibited moderate antibacterial effects, with inhibition zones observed only for isolate PS 2 (10 ± 0.61 mm) and MIC values of 1–2 mg/mL across the isolates. Enhanced antibacterial activity was recorded for IgY–Poly/ChNPs, which produced inhibition zones ranging from 12 ± 0.98 to 20 ± 0.63 mm and MIC values between 0.5 and 1 mg/mL, indicating improved growth inhibition following IgY conjugation. Notably, IgY-coated Poly/ChNPs demonstrated the strongest antibacterial activity among all tested formulations. This formulation produced the largest inhibition zones (16 ± 0.97 and 21 ± 0.91 mm) and the lowest MIC values (0.125–0.5 mg/mL), highlighting an additional enhancement in antibacterial potency. Isolate PS 2 was the most susceptible, exhibiting the lowest MIC value (0.125 mg/mL), whereas PS 1 and PS 3 showed comparatively higher MICs. Overall, the MIC results corroborate the inhibition zone findings, as formulations producing larger zones of inhibition were consistently associated with lower MIC values. These data confirm that nanoparticle formulation and IgY surface modification significantly improve antibacterial effect against colistin-intermediate and resistant isolates, while also demonstrating isolate-dependent variability in susceptibility. Chitosan, a naturally occurring polycationic polysaccharide, was selected as a coating material because of its biocompatibility, biodegradability, mucoadhesive capacity, and intrinsic antibacterial activity. Its mucoadhesive properties may enhance the retention of colistin-loaded nanoparticles at the target site, thereby prolonging drug residence time, supporting sustained antibiotic release, and improving local absorption. These characteristics may permit reductions in both dosage and dosing frequency. Moreover, combining chitosan with colistin may produce a synergistic antibacterial effect, as has been reported for other antibiotic agents.

**Table 5:** Screening of minimum inhibitory concentrations (MICs) and inhibition zone diameters of the tested compounds against colistinnon-susceptible *Salmonella* Typhimurium isolates.

Bacterial isolate	Inhibition zone diameter (mm) (mean± SD)				
	IgY	IgY/ChNPs	Poly/ChNPs	IgY-Poly/ChNPs	IgY-coated-Poly/ChNPs
**PS 1**	0	0	0	18±0.84	21±0.91
**PS 2**	0	0	10±0.61	20±0.63	20±0.72
**PS 3**	0	0	0	12±0.98	16±0.97

Bacterial isolate	MIC values (mg/mL) (mean± SD)				
**PS 1**	>2	>2	1	0.5	0.25
**PS 2**	>2	>2	1	0.5	0.125
**PS 3**	>2	>2	2	1	0.5

### Time–kill assay

**Fig. 5:**
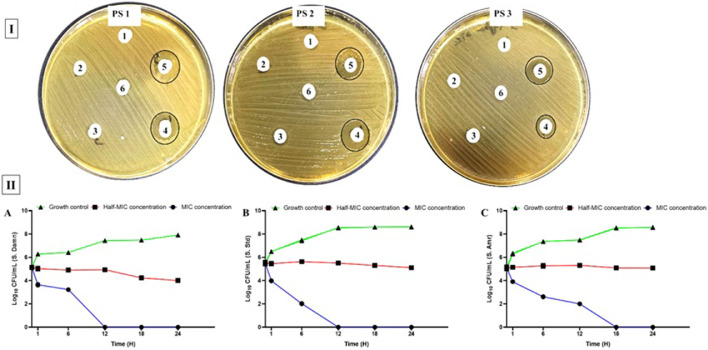
(I) Agar diffusion and inhibitory effect of IgY-polymyxin B-based NPs against colistin-non-susceptible *Salmonella* Typhimurium isolates. 1 IgY; 2 IgY/ChNPs; 3 Poly/ChNPs; 4 IgY-Poly/ChNPs; 5 IgY-coated-Poly/ChNPs; and 6 sterile distilled water (negative control). (II) Curves represents the time killing assays of colistin-non-susceptible *Salmonella* Typhimurium isolates after treatment with IgY-coated-Poly/ChNPs.

The time–kill approach was employed to characterize the dynamic antibacterial activity of the tested formulation over time and to distinguish between bacteriostatic and bactericidal effects. The colistin-non-susceptible *Salmonella* Typhimurium isolates were exposed to the tested formulations at doses equivalent to the MIC and half the MIC (½ × MIC). As illustrated in Fig. 5(II), untreated cultures exhibited progressive growth throughout the 24-h incubation period, reflecting normal replication kinetics. In contrast, treated cultures showed a marked suppression of bacterial growth that was dependent on both concentration and exposure time. At ½ × MIC, bacterial counts remained relatively stable over the incubation period, with no notable increase in colony-forming units (CFU), indicating effective growth inhibition without cell killing. At the MIC level, a pronounced decline in CFU was observed shortly after treatment initiation. For PS 2, bacterial counts decreased rapidly, reaching the lowest levels within approximately 12 h, whereas PS 3 exhibited a more gradual reduction, with maximal killing observed around 18 h. These findings indicate that exposure at the MIC resulted in sustained bacterial killing relative to the initial inoculum.

### Expression of the mcr-1 gene under polymyxin B and nanocombinations treatments

**Fig. 6:**
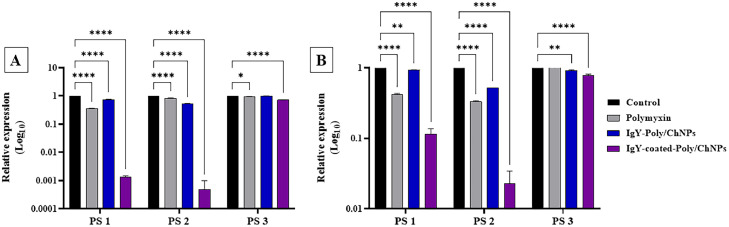
Quantitative real-time PCR analysis showing the expression of the *mcr-1* gene in colistin-non-susceptible *Salmonella* Typhimurium isolates. (**A**) and (**B**) represent relative fold changes obtained using mcr-1 and M1 primers, respectively, from two independent qPCR assays. Data are presented as mean ± SD. Statistical significance relative to the untreated control is indicated as *P < 0.05, **P < 0.01, ***P < 0.001, and ****P < 0.0001.

The plasmid-mediated mcr-1 gene is recognized as one of the principal determinants of transferable resistance to colistin among Gram-negative bacteria (Doumith et al., 2016; Ngbede et al., 2020). Since its first description by Liu Yongning and colleagues in 2015, the gene has been reported across multiple geographic regions and in isolates from animals, food sources, and humans, highlighting its importance in the epidemiology of antimicrobial resistance (Liu et al., 2016; Nagy et al., 2021). The mcr-1 gene encodes a phosphoethanolamine transferase that modifies lipid A within lipopolysaccharide (LPS), decreasing the negative charge of the bacterial outer membrane and thereby reducing colistin binding affinity (Feng et al., 2022). This structural modification preserves membrane integrity and contributes directly to phenotypic resistance (Zhu et al., 2021). The presence and transcriptional response of the mcr-1 gene were investigated in the colistin-non-susceptible *Salmonella* Typhimurium isolates using quantitative real-time PCR. As shown in Fig. 6, all tested isolates were confirmed to contain the mcr-1 gene, with baseline expression levels normalized to the untreated controls. Exposure to polymyxin B at sub-inhibitory concentrations resulted in either maintenance or slight upregulation of mcr-1 expression across the isolates. In PS 1- and PS 2, polymyxin B treatment led to a reduction relative to the control but remained within the same order of magnitude, whereas PS 3 showed expression levels comparable to untreated cells. These findings suggest that polymyxin B exposure does not suppress mcr-1 transcription and may contribute to sustained resistance-associated gene expression. Treatment with IgY–Poly/ChNPs produced isolate-dependent effects on mcr-1 expression. While moderate downregulation was observed in PS 1 and PS 2, expression levels in PS 3 remained largely unchanged compared with the control, indicating limited transcriptional suppression in this isolate. In contrast, IgY-coated-Poly/ChNPs caused a pronounced downregulation of mcr-1 expression in PS 1 and PS 2, with transcript levels decreasing by approximately three orders of magnitude relative to untreated controls. This marked suppression was not observed in PS 3, where mcr-1 expression remained comparatively high, suggesting isolate-specific variability in transcriptional response to treatment. The observed suppression of mcr-1 expression may be attributed to the multifunctional design of the IgY–coated–Poly/ChNPs. Chitosan has been reported to disrupt outer membrane integrity and alter surface permeability, while colistin provides localized antimicrobial pressure (Dubashynskaya et al., 2023). In addition, IgY may interfere with bacterial surface structures and reduce cellular fitness and adhesion capacity. Together, these effects may compromise membrane remodelling pathways and reduce the selective advantage associated with mcr-1 expression. Because LPS is essential for maintaining the structural stability and barrier function of Gram-negative bacteria, interference with lipid A modification pathways may weaken bacterial defenses and restore susceptibility to polymyxins B (Gao et al., 2016). The reduction in mcr-1 expression observed following nanoparticle treatment suggests that these formulations may help reverse plasmid-mediated colistin resistance while enhancing antibacterial efficacy (Cho et al., 2021; Ravi et al., 2025).

### Antibiofilm assessment

**Fig. 7:**
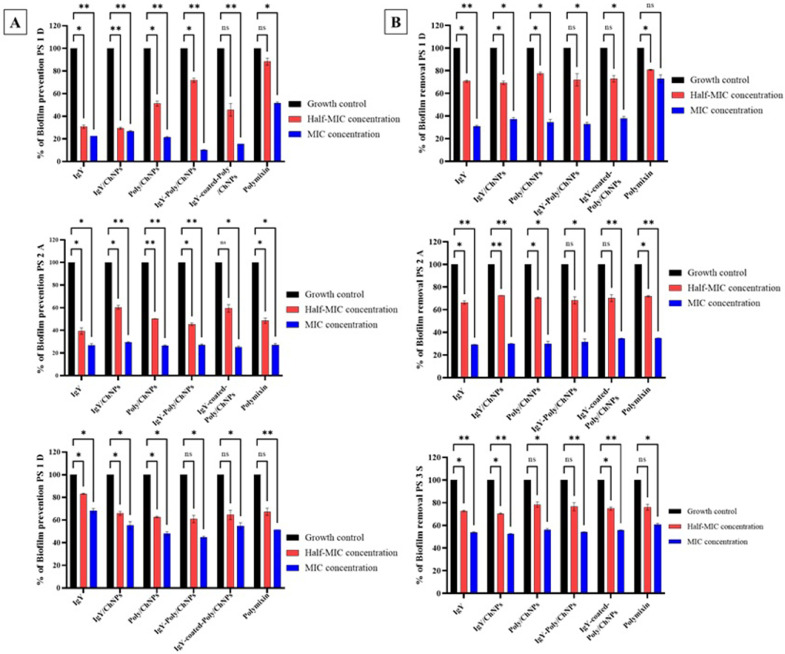
Percent of formed biofilm (**A**) Inhibitory effect of the tIgY-polymyxin B-based NPs on preventing the biofilm formation. (**B**) Disruptive effect of the IgY-polymyxin B-based NPs on mature biofilms. Data are presented as mean ± SD. Statistical significance relative to the untreated control is indicated as *P < 0.05, **P < 0.01, ***P < 0.001, and ****P < 0.0001.

As shown in Fig. 7 (A and B) represent the microscopic observations and quantitative analysis, respectively, illustrating the effect of the tested nanocombinations on the prevention of biofilm formation by the bacterial isolates. Almost all tested formulations resulted in a reduction in biofilm formation compared with the untreated control. The antibiofilm activity was more pronounced at the MIC than at half the MIC, indicating a concentration-dependent inhibition of biofilm formation. The biofilm prevention effect was more evident in PS 1 and PS 2 than in PS 3. This difference may be attributed to the colistin-resistant phenotype of PS 3, whereas the other isolates showed intermediate susceptibility. Surface charge of nanoparticles displays a significant role in antimicrobial performance. The antibacterial effect of chitosan is largely linked to electrostatic interactions between its positively charged amino groups and negatively charged bacterial cell wall, which can increase membrane permeability and cause leakage of intracellular components (Li and Zhuang, 2020). Consequently, negatively charged chitosan–dextran sulfate systems may exhibit weaker direct electrostatic interactions with bacterial membranes. However, these systems often demonstrate improved colloidal stability and can facilitate controlled drug release and enhanced penetration into biofilm matrices, which may compensate for reduced charge-mediated membrane disruption (Chen et al., 2007).

The biofilm prevention percentages achieved by the final formulation (IgY-coated Poly/ChNPs) ranged from 35% to 85%, with the highest inhibition observed in PS 1 and the lowest in PS 3. The ability of the tested formulations to disrupt pre-formed biofilms is presented in Fig. 7 (C and D). Treatment of mature biofilms at the MIC and half MIC resulted in biofilm reduction ranging from 40% to 70% for most tested samples. Notably, biofilm prevention was more effective than the removal of established biofilms under the same experimental conditions. The persistence of *Salmonella* Typhimurium in poultry production systems is strongly associated with its capacity to form biofilms, which enhances environmental survival, facilitates transmission, and contributes to reduced antimicrobial susceptibility (Rossi et al., 2017; Avcı et al., 2026). In this study, two experimental approaches were employed: prevention of biofilm formation through co-incubation of bacterial suspensions with the tested formulations for 48 h, and disruption of pre-established biofilms.The preventive approach yielded markedly stronger inhibition than the removal of mature biofilms, indicating that the formulations are more effective at interfering with early adhesion and biofilm development than at dismantling established biofilm structures.

Previous studies indicate that colistin can reduce biofilm formation at sub-MIC concentrations; however, sub-inhibitory exposure may also produce variable or even stimulatory effects depending on bacterial strain and experimental conditions. Nano-delivery systems have been shown to enhance colistin performance by improving localization and sustained release. For example, colistin-loaded human albumin nanoparticles demonstrated significantly enhanced inhibition of biofilm formation compared with free colistin, achieving up to 4-fold and 60-fold greater inhibition in susceptible and resistant strains, respectively (Scutera et al., 2021). Furthermore, recent nanoparticle-based antibiotic systems have shown improved antibiofilm efficacy and reduced MIC values compared with free drugs, supporting the advantage of nano-enabled delivery in combating resistant Gram-negative pathogens (Şirin et al., 2023). The nanoformulations developed in this study, composed of chitosan nanoparticles loaded with colistin and incorporating or coated with IgY using dextran sulfate, exhibited a net negative surface charge. Despite this charge reversal, the formulations retained strong antibiofilm activity, suggesting that mechanisms beyond electrostatic membrane interaction are involved.

Chitosan may interfere with early bacterial adhesion and matrix assembly, colistin provides localized antimicrobial action, and IgY may contribute to blocking bacterial surface structures required for attachment and early biofilm establishment. Notably, nanoparticles incorporating IgY and polymixin demonstrated antibiofilm activity comparable to, and in some cases exceeding, that of IgY-coated formulations, indicating that incorporation within the nanoparticle matrix may enhance functional interaction with bacterial cells during early biofilm development. Variability in biofilm modulation by colistin and nano-delivery systems has been widely reported and is influenced by antimicrobial mechanism, bacterial strain, resistance profile, and concentration range (Ahsan et al., 2024). Sub-MIC exposure can produce non-linear responses due to complex regulatory pathways governing biofilm formation. Additionally, negatively charged nanoparticles have been reported to penetrate mucus and biofilm matrices effectively, enabling improved delivery of antimicrobial payloads and enhanced biofilm eradication. The enhanced activity observed in this study therefore likely arises from combined effects of improved drug localization, penetration into the developing biofilm matrix, and interference with bacterial attachment processes rather than surface charge–dependent membrane disruption alone (Yu et al., 2024).

### SEM images analysis

**Fig. 8:**
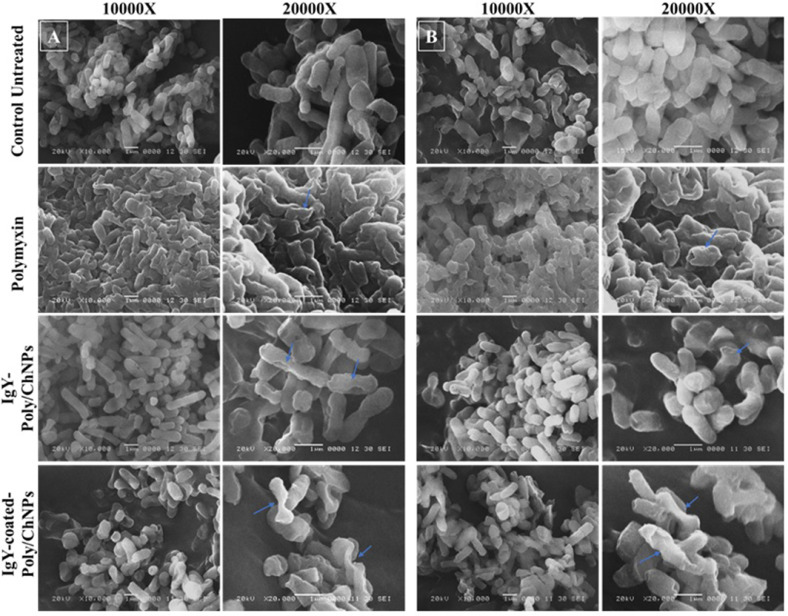
Scanning electron microscopy (SEM) micrographs of two bacterial isolates (**A**) PS 2 and (**B**) PS 3. Images were captured at magnifications of 10,000 × (left panels) and 20,000 × (right panels). The blue arrows indicate cell shrinkage, membrane roughening, and localized lysis.

As shown in Fig. 8A, the untreated PS 2 isolate (colistin-intermediate resistant) maintained its typical rod-shaped morphology with smooth and intact cell surfaces. The majority of cells appeared structurally preserved, with no obvious signs of membrane damage. After treatment, the degree of structural alteration varied depending on the formulation used. Cells treated with colistin alone showed very limited morphological changes, supporting the intermediate resistance profile of this isolate. Treatment with IgY-Poly/ChNPs caused mild surface irregularities and slight deformation in some cells; however, most bacteria retained their general structure. The most noticeable effect was observed following treatment with IgY-coated-Poly/ChNPs. As illustrated in Fig. 8A, several cells exhibited clear signs of cell wall disruption, shrinkage, and surface roughening. Moderate deformation and membrane irregularities were evident, suggesting significant compromise of the bacterial cell wall. In the case of PS 3 (colistin-resistant isolate), the untreated cells also showed intact morphology, as presented in Fig. 8B. Following treatment, structural changes were less pronounced compared to PS 2. Although IgY-coated-Poly/ChNPs induced observable surface irregularities and partial deformation in some cells, the overall impact was comparatively reduced. Notably, colistin treatment alone produced minimal morphological changes in PS 3, confirming its resistant phenotype. Similarly, IgY-Poly/ChNPs demonstrated limited structural disruption. Nevertheless, IgY-coated-Poly/ChNPs still showed a greater effect than either colistin or IgY-Poly/ChNPs alone. In this study, neither free IgY nor IgY/ChNPs produced measurable inhibition zones or detectable MIC values, confirming that polymyxin B remains the primary bactericidal component within the formulations. However, the IgY-coated Poly/ChNPs demonstrated the most potent antibacterial activity, suggesting that the IgY coating and chitosan matrix potentiate the antibiotic's effect, likely by improving stability or facilitating closer interaction with the bacterial membrane.

## Conclusion

The designed IgY-polymyxin B-based NPs exhibited well-defined core–shell architecture with spherical or semi-spherical morphology and negligible aggregation with improved colloidal stability and thermal resistance. In vitro antibacterial investigations demonstrated that the polymyxin B-loaded nanocombinations, enhanced by IgY coating, have potent efficacy against multidrug-resistant *Salmonella* Typhimurium isolate. Time–kill kinetic analysis confirmed a bactericidal effect that is both dose- and time-dependent. Furthermore, these formulations were shown to suppress the expression of the mcr-1 gene and inhibit biofilm formation while displaying minimal cytotoxicity toward normal human cell lines. Collectively, these results highlight the potential of these nanocombinations as candidates for future antimicrobial therapy. However, while these in vitro findings are promising, further in vivo validation in animal models is required to assess therapeutic efficacy, pharmacokinetics, and safety in a living system before clinical applications can be considered.

## Contributions

Each author declares substantial contributions through the following:

(1) the conception and design of the study, or acquisition of data, or analysis and interpretation of data, (2) drafting the article or revising it critically for important intellectual content,

Please indicate for each author the author contributions in the text field below. Signatures are not required.

## Approval of the submitted version of the manuscript

Please check this box to confirm that all co-authors have read and approved the version of the manuscript that is submitted. Signatures are not required.

## CRediT authorship contribution statement

**Ehab H. Mattar:** Writing – original draft, Validation, Software, Resources, Formal analysis, Data curation. **Ali T. Zari:** Writing – original draft, Validation, Software, Resources, Investigation, Formal analysis. **Esmail M. El-Fakharany:** Writing – original draft, Visualization, Validation, Software, Resources, Methodology, Investigation, Formal analysis, Data curation. **Yousra A. El-Maradny:** Writing – original draft, Visualization, Validation, Software, Resources, Methodology, Investigation, Formal analysis, Data curation. **Bassam O. Aljohny:** Validation, Software, Resources, Data curation. **Elrashdy M. Redwan:** Writing – review & editing, Visualization, Supervision, Project administration, Funding acquisition, Data curation, Conceptualization.

## Disclosures

The authors declare that they have no known competing financial interests or personal relationships that could have appeared to influence the work reported in this paper.
